# Effect of the Structural and Morphological Properties of Surfactant-Assisted Hydroxyapatite on Dermal Irritation and Antibacterial Activity

**DOI:** 10.3390/ma14216522

**Published:** 2021-10-29

**Authors:** Giovanni García Domínguez, Sebastián Diaz De La Torre, Lorena Chávez Güitrón, Erasto Vergara Hernández, Joan Reyes Miranda, Maribel Quezada Cruz, Aristeo Garrido Hernández

**Affiliations:** 1 Instituto Politécnico Nacional, CIITEC IPN, Cerrada de Cecati S/N, Col. Santa Catarina, Azcapotzalco, Ciudad de México 02250, Mexico; garciagiovanni1311@gmail.com (G.G.D.); sediazt@ipn.mx (S.D.D.L.T.); 2 Universidad Tecnológica de México—UNITEC MÉXICO–Campus Ecatepec, Ecatepec de Morelos 55107, Estado de México, Mexico; lrnchavez@yahoo.com; 3 Universidad Tecnológica de Tecámac, UTTEC, Carretera Federal México–Pachuca Km 37.5, Col. Sierra Hermosa, Tecámac 55740, Estado de México, Mexico; mabelqz@yahoo.com.mx; 4 Instituto Politécnico Nacional, UPIIH, Carretera Pachuca—Actopan Kilómetro 1+500 Ciudad del Conocimiento y la Cultura, San Agustín Tlaxiaca 42162, Hidalgo, Mexico; erasto99@hotmail.com; 5 Universidad Autónoma Metropolitana, UAM, Av. San Pablo Xalpa 180, Col. Reynosa-Tamaulipas, Azcapotzalco, Ciudad de México 02200, Mexico; joremi@azc.uam.mx

**Keywords:** hydroxyapatite, nanorods, antibacterial activity, hydrothermal, surfactant

## Abstract

Hydroxyapatite (HAp) nanoparticles with a homogeneous rod morphology were successfully synthesized using the hydrothermal method. The powders were characterized using Fourier transform infrared spectroscopy, X-ray diffraction, and scanning electron microscopy. The antibacterial and dermal irritation analyses of the samples were performed and discussed. The use of cationic and anionic surfactants, namely, cetyltrimethylammonium bromide (CTAB) and sodium dodecyl sulfate (SDS), respectively, at a low concentration (2.5 mol%) modified the length/diameter (L/D) ratio of the HAp rods. Structural characterizations of hydroxyapatite synthesized without surfactant (HA), with 2.5 and 5 mol% of SDS (SDS− and SDS+, respectively), and with 2.5 and 5 mol% of CTAB (CTAB− and CTAB+, respectively) revealed well-crystallized samples in the hexagonal phase. The CTAB− sample presented antibacterial activity against *Pseudomonas aeruginosa*, *Escherichia coli*, *Streptococcus anginosus*, *Staphylococcus aureus*, *Micrococcus luteus*, and *Klebsiella pneumoniae*, suggesting that antimicrobial susceptibility was promoted by the bacterial nature and the use of the surfactant. Dermal irritation showed no clinical signs of disease in rabbits during the study, where there was neither erythema nor necrosis at the inoculation sites.

## 1. Introduction

In orthopedic surgery, hydroxyapatite (Ca_10_ (PO_4_)_6_(OH)_2_) is the most used material due to its highly similar chemical composition to the inorganic part of the human bone and stability under physiological conditions [1,2]. Hydroxyapatite (Hap) has been used as a bio-coating on metal implants in the human body, offering high biocompatibility; it has a wide range of applications regarding bone tissue engineering, dental prostheses, thin-film coating, bone defects, and bone replacements. In addition to their use in bone-related engineering, HAp-based materials offer a wide range of biological applications, such as drug delivery, protein cleavage, and gene transfer, mainly due to their benign nature [3,4,5]. However, for all these uses, like other biomaterials, the studies on skin irritation are important for evaluating biological safety, which validated the potential application of HAp powders.

In this vein, the design of materials for preventing implant-associated infections has been a relevant issue recently [6] since infections due to medical implants and injection materials are still very common [7,8].

Microbial pollution produced by microorganisms has contributed to numerous problems in human life. In particular, osteomyelitis, which is usually caused by *Staphylococcus aureus* (*S. aureus*), causes many complications regarding trauma and orthopedic surgery [9]. Infections in living tissues that are caused by Gram-positive and Gram-negative bacteria can occur mainly when foreign material comes into contact with the body, e.g., in surgical procedures, and can trigger some kind of microbial processes and cause an infection in the contact area of the body. Despite the chemical and structural similarities, the bioactivity and mechanical capabilities of synthetic hydroxyapatite are still far inferior to those of natural hard tissues. Therefore, the fabrication of HAp composite materials with antibacterial properties becomes a good solution. Thus, the design of HAp particles for this purpose was possible using different synthesis methods. For obtaining specific morphologies and particle sizes, wet chemical methods were used [10]. The hydrothermal method is considered a promising approach for the large-scale and low-cost production of HAp particles; it presents many advantages, such as chemical homogeneity, large crystal production with high quality, and is catalyst-free [11]. Furthermore, surface-active agents (surfactants) play an important role in aqueous hydrothermal solutions because their hydrophobic and hydrophilic groups change the surface charge of particles, modifying the properties of the nanoparticles [12,13]. Surfactants are widely used in different industries and laboratories for getting nanoparticles with tunable morphologies and different particle sizes [14].

In this regard, a cationic surfactant, namely, cetyl trimethyl ammonium bromide (CTAB), and an anionic surfactant, namely, sodium dodecyl sulfate (SDS), were used to regulate nucleation and crystal growth. Gopi et al. synthesized nano-sized HAp particles with different growth modifications, such as nano-sized spheres, rods, and fibers, via a microwave coupled hydrothermal method using CTAB as a template [15]. Furthermore, Yan et al. obtained fibrous polycrystals of HA at room temperature and, after hydrothermal treatment and using SDS, they were transformed into nanorods of 150 nm in length and 10 nm in diameter [1]. HAp nanoparticles with nanorod, nanowire, and nanotube morphologies showed excellent biocompatibility [16]. Yingjun et al. synthesized HAp nanoparticles with uniform morphologies and controllable size using a low-temperature hydrothermal process. In addition to the morphology, crystallographic characterization is an important issue for biomaterials [17]; normally, these features are elucidated using the X-ray diffraction technique. For instance, the Scherrer equation predicts the crystallite size (smaller than 100 nm). It is well-known that instrumental broadening affects the crystallite size, which must be corrected by identifying the broadening contribution of the instrument. It was reported that the crystallite size and crystallinity affect the antibacterial capacity [18] and bioactive properties [19] of HAp. Rabiei et al. stated that the crystal size of HAp in animal bones is important for doping metals, bioglass, polymers, and implant applications [20].

At the nanoscale, the study of the bacteria–nanoparticle interactions is very important because of the surface–volume ratio. On the other hand, antibacterial activity mechanisms have not been fully elucidated; therefore, more studies concerning ceramic nanoparticles should be performed.

In this work, HAp nanorods with different length/diameter ratios were prepared using CTAB and SDS as cationic and anionic surfactants, respectively. The antibacterial activity of the powders was tested on six pathogenic bacteria: *Pseudomonas aeruginosa*, *Escherichia coli*, *Streptococcus anginosus*, *Staphylococcus aureus*, *Micrococcus luteus*, and *Klebsiella pneumoniae.* To assess the dermal irritation that was caused by the HAp powders, young male New Zealand rabbits were employed. The results indicated that the antibacterial and dermal irritation properties depended on the length/diameter (L/D) ratio of the HAp particles.

## 2. Materials and Methods

### 2.1. Hydroxyapatite Synthesis

In the HAp syntheses, calcium nitrate hexahydrate CaNO_3_·6H_2_O (Mallinckrodt 99%, Staines, UK), di-ammonium hydrogen phosphate (NH_4_)_2_HPO_4_, (Baker Analyzed^TM^, 98.7%, Radnor, Pennsylvania, US) were used in the hydrothermal method as starting materials. Cetyltrimethylammonium bromide (CTAB, ACS reagent, ≥99.0%, Sigma Aldrich Corporation, St. Louis, Missouri, US) and sodium dodecyl sulfate (SDS, ReagentPlus, ≥98.5%, Sigma Aldrich Corporation, St. Louis, MO, USA) were used as surfactants. (NH_4_)_2_HPO_4_ solution at 0.5 M was added dropwise to a 0.5 M CaNO_3_·6H_2_O solution. The cationic surfactant was added to the Ca^2+^ solution or the anionic one to the PO_4_^3−^ solution. During the addition process, the Ca^2+^ solution was kept under stirring at 50 °C for 15 min. Then, the final solution was adjusted to pH = 11 using a NaOH solution (1.5 M). The reaction volume was fixed at 32 mL and placed in the Teflon^®^-lined autoclave (Parr instrument, Illinois, US, capacity 45 mL) at 200 °C for 15 h. The obtained powders were washed with distilled water using a centrifuge (Solbat, Puebla, México, 10,000 rpm, 15 min each wash). Finally, these powders were dried in an oven (Faithful Instrument, Huanghua, CN) at 120 °C for 8 h. For better identification, the prepared hydroxyapatite samples were named HA (without surfactant), SDS− and SDS+ (synthesized at 2.5 and 5 mol% of SDS), and CTAB− and CTAB+ (synthesized at 2.5 and 5 mol% of CTAB).

### 2.2. Characterization Techniques

The X-ray diffraction patterns were recorded at room temperature on a Bruker D8 Advance Eco diffractometer (Bruker, Billerica, MA, USA) using Cu Kα radiation (λ = 1.54184 Å). To determine the crystalline phase of the samples, a 2θ scan ranging from 10 to 60° with scanning conditions of a 0.4 slit and a 0.02° step was used. A total of 0.5 g of dry powder was analyzed for each sample; under the same conditions, two additional measurements with independent preparation within a range of 2θ = 25–35° were performed. All the data were collected using the DIFFRAC.SUITE™ software (Bruker, Billerica, Massachusetts, US). Infrared spectra were recorded on a Perkin Elmer Spectrum Two Spectrophotometer (PerkinElmer, Waltham, Massachusetts, USA) using the ATR mode. The spectrum was recorded using 32 scans in the 4000–500 cm^−1^ range. The SEM micrographs were recorded using a JOEL scanning electron microscope model JSM-78000F (Jeol Ltd Akishima, Tokyo, Japan) operating under a high vacuum and a low accelerating voltage of 5 kV. The length and diameter distribution were obtained by measuring the length and diameter of 100 particles for each sample. The particles were selected from different images at 4000X and different areas per sample. To facilitate the particle distribution, an image processing program, namely, ImageJ, was used. The statistical significance of the length and diameter of each sample was determined using one-way analysis of variance (ANOVA) with Minitab, where *p* < 0.05 represented statistical significance. After that, a post hoc Tukey’s HSD (honestly significant difference) test was performed. Finally, the length/diameter ratio was obtained using those values from the length and particle distribution since the measurements were obtained from a particular rod. All the characterization data were plotted using OriginPro 8.1 software (OriginLab, Northampton, MA, USA).

### 2.3. Antibacterial Activity

The antibacterial activities of the HA, SDS−, and CTAB− were tested on six pathogenic bacteria, where three of them were Gram-negative—*Pseudomonas aeruginosa* CDBB-B-1011, *Escherichia coli* CDBB-B-1000, and *Streptococcus anginosus* CDBB-B-1311—and three were Gram-positive—*Staphylococcus aureus* CDBB-B-1304, *Micrococcus luteus* ATCC 10240, and *Klebsiella pneumoniae* ATCC10031—which were obtained from the National Collection of Microbial Strains and Cell Cultures at the Research Center for Advanced Studies (CINVESTAV-IPN), Mexico, Mexico.

Before the antibacterial activity test, the strains were cultivated on plates with nutritive agar and incubated at 35 ± 1 °C for 24 h. The colonies were re-suspended in sterile distilled water until obtaining a turbidity value of 0.125 (McFarland turbidity standard) corresponding to 1 × 10^8^ CFU/mL. The antibacterial activity was determined using the Kirby–Bauer test [21]. Three sensitive discs of 6 mm in diameter were placed equidistant to each other in the three plates: blank (plate with culture medium only), positive, and negative controls. The positive control consisted of a plate with a culture medium, bacterial inoculum, and antibiotic in a commercial sensitive disc (tetracycline), while the negative control only contained bacterial inoculum and the culture medium. For the test, sen-sitive discs were introduced into 0.5 mg/mL of HA, SDS−, or CTAB− solution for one minute.

Three sensitive discs of 6 mm in diameter were placed equidistant to each other in the three plates. A control (plate with culture medium only) and two controls; positive and negative.

### 2.4. Dermal Irritation Procedure

Young male New Zealand rabbits in the 1.8–2.2 kg range were used. These rabbits were monitored for 14 days in their new environment (temperature and humidity). After this period, rabbits with good health were selected for the dermal irritation assays. Commercial feed and water were supplied ad libitum (as much or as often as necessary or desired) during the study. The animals were kept alone in a well-identified stainless-steel cage. Rabbits were shaved in the dorsal region (6 cm × 12 cm) using an electric razor.

An amount of 0.5 mg of hydroxyapatite powders was dispersed in one mL of sterile physiological saline solution. Before inoculation, the shaved area was disinfected using alcohol. In the upper region, on the right and left side, an adjuvant (casein 3.5 g/100 mL and lactose 5 g/100 mL) was administered (positive control). The hydroxyapatite solution was applied in the shaved central region, along with the physiological saline solution (negative control). One milliliter of HAp solution or a negative or positive control was used for the intradermal inoculation. The presence of erythema, edema, induration, or necrosis was evaluated at 1, 24, 48, and 72 h after inoculation.

Laws and institutional guidelines were considered according to the Mexican official norms for carrying out the experimentation, transportation, and care of the animals in this study. NOM-051-Z00-1995 (Humane treatment in the transportation of animals), NOM-087-ECOL-1995 (Requisites for the separation, packaging, storage, collection, transportation, treatment, and final disposal of biological infectious hazardous waste generated in establishments providing medical care), and NOM-062-Z00-1999 (Technical specifications for the production, care, and use of laboratory animals).

## 3. Results

### 3.1. Structural and Morphological Characterization

**Figure 1:**
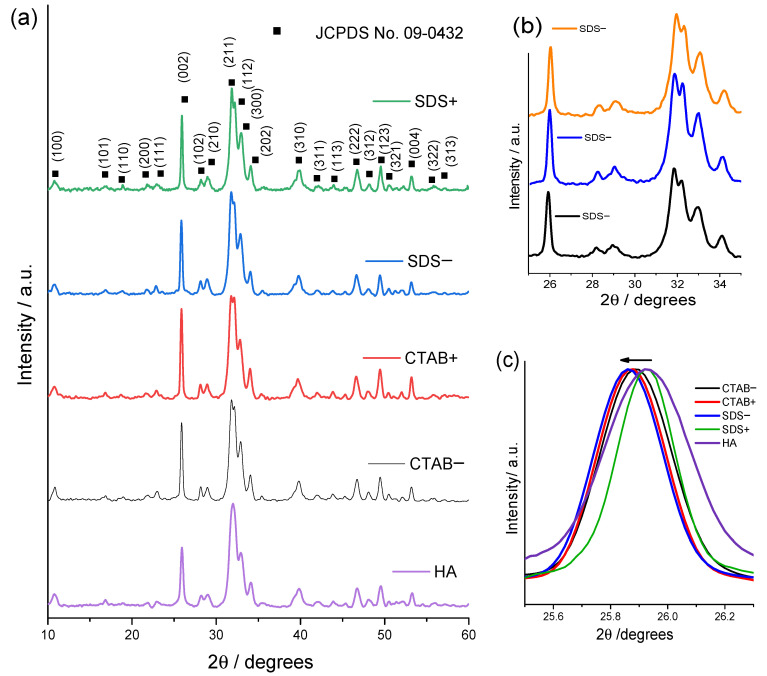
(**a**) XRD patterns of HA, CTAB−, CTAB+, SDS−, and SDS+ samples; (**b**) repeated measurements of the SDS−sample within a range of 2θ = 25–35°; and (**c**) shift of the peak resulting from (002) plane.

Figure 1 displays the X-ray diffraction (XRD) patterns of the HAp samples. All the samples crystallized with the hexagonal structure of hydroxyapatite (JCPDS no. 09-0432) [22]. Thus, the surfactants that were used during the synthesis did not change the crystal structure; however, the main diffraction peaks presented a difference in their relative intensities, especially for the (002) and (211) planes. Therefore, the I_002_/I_211_ ratio changed as a function of the surfactant concentration and type. The I_002_/I_211_ ratios for samples HA, CTAB+, CTAB−, SDS+, and SDS− were 0.639 ± 0.073, 0.823 ± 0.048, 0.763 ± 0.024, 0.8 ± 0.062, and 0.739 ± 0.025 (see Table 1), respectively. The standard deviation was obtained from three repeated measurements (for reliability, see Figure 1b). The intensity change could be related to a preferential orientation that is produced by the length/diameter ratio of the rods.

According to the literature, the crystallinity of hydroxyapatite can be elucidated using the full width at half maximum (FWHM) of the (002) reflection [23]. Many findings pointed out that the sharper the XRD reflection, the bigger crystallite size and the better the crystallinity; for instance, the sharp and strong intensity of the XRD peaks indicated a highly crystallized anhydrous dicalcium phosphate (DCPA) [24].

The crystallite size of the HAp powders was determined using the Scherer equation [25]:

*d= (K λ)/(β cosθ)* (1)

where *d* is the average crystallite size, *λ* = 0.154184 nm is the X-ray radiation, *β* is the peak width of the diffraction peak profile at half maximum height in radians, and *K* is a constant that is related to the particle shape, which is normally taken to be 0.94.

Since the instrumental broadening (*β*) modifies the crystallite size of nanoparticles estimated by the Scherrer formula, the broadening contribution of the instrument should be subtracted. Therefore, the diffraction pattern of the standard silicon material was employed [26,27]. The corrected FWHM, namely, $\beta_{hkl}$, was calculated using Equation (2):

(2)  βhkl=[βhkl2measured−β2instrumental ]12 

where $\beta_{measured}$ is the FWHM of the (002) reflection measured and $\beta_{instrumental}$ is the instrumental broadening.

Figure 1c shows a clear difference in FWHM between the samples; the broader peak corresponds to the HA sample, while the narrower peak corresponds to the SDS+ sample. The crystallite size varied from 23.827 ± 1.65 nm for the HA sample to 47.3 ± 1.188 nm and 34.027 ± 1.308 nm for SDS+ and CTAB+, respectively.

The lattice parameters (*a* and *c*) were calculated using the Bragg equation and the lattice geometry (Equations (3) and (4)) based on the HCP standard unit cell plane and the spacing relationship from (002) and (211) reflections [22,28]:

(3) $$\lambda =2d_{hkl}sin\theta \;$$

(4) $$\frac{1}{d_{hkl}{}^{2}}=\frac{4}{3}\left(\frac{h^{2}+hk+k^{2}}{a^{2}}\right)+\frac{l^{2}}{c^{2}}$$

Figure 1c shows that the peak located around 2θ = 25.92° corresponding to the (002) reflection, which was slightly shifted toward lower 2θ values when surfactants were used, suggesting that the lattice parameter of the hexagonal structure increased; this tendency was observed in the values of the lattice parameter a and b (see Table 1).

This peak also became narrower with the use of surfactants (see Figure 1c). It was reported in the literature that wider and narrower XRD peaks indicate lower and higher crystallinity in hydroxyapatite powders, respectively [29,30]. Table 1 shows that the crystallite size increased with the addition of surfactants. The higher the CTAB or SDS concentration, the larger the crystallite size. This suggests that small quantities of CTAB or SDS could affect the crystallite size of HAp. A similar finding was reported by Yang et al. [31], where they found that the average crystallite sizes of luminescent phosphates synthesized by precipitation method increased when the PEG-20000 (polyethylene glycol with a molecular weight of 20,000 g/mol, classified as nonionic surfactant) concentration increased. Gopi et al. also reported that by increasing the amount of CTAB, the crystallite size and crystallinity increased [15]. In this study, the use of CTAB and SDS as surfactants directly affected the crystallite size.

**Figure 2:**
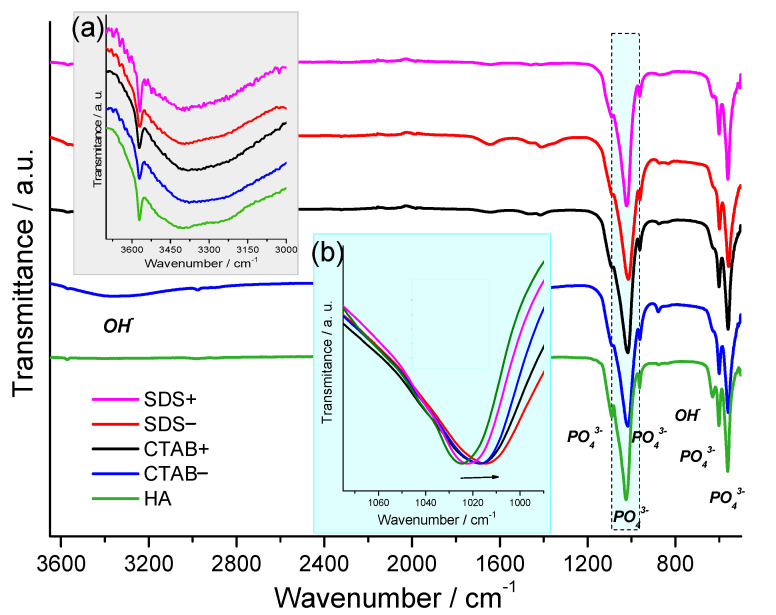
Infrared spectra of HA, CTAB−, CTAB+, SDS−, and SDS+ samples. Inset (**a**): the 3700–2000 cm^−1^ region of infrared spectra of the HAp samples; inset (**b**): shift of the absorption band located around 1025 cm^−1^.

Figure 2 shows the spectra using attenuated total reflectance–Fourier-transform infrared (ATR–FTIR) spectrometry of the hydroxyapatite powders that were synthesized using the CTAB− and SDS-assisted hydrothermal method to evaluate the effects of the surfactants; HAp powder that was synthesized using the free-surfactant hydrothermal method was also included. The infrared spectra exhibited a similar band between the samples and no absorption bands corresponding to the surfactants were found; thus, they were effectively removed in the washing stage. The broad FTIR band centered approximately at 1025 cm^−1^ corresponded to the asymmetric stretching vibration mode of the PO_4_^3−^ group. The band at 550 cm^−1^ corresponded to the symmetric P–O stretching vibration of the PO_4_^3−^ group. The absorbance in ATR mode diminished toward higher wavenumbers [32]; consequently, the OH− vibration characteristic located around 3570 cm^−1^ of apatite was not evident in the infrared spectra. Thus, a magnification of the infrared spectra of the samples inset (a) in Figure 2 is presented. In the inset (a), it is clear the characteristic absorption band of HAp was related to O–H stretching vibration located around 3570 cm^−1^; in addition, Figure 2 shows the band located around 630 cm^−1^, which was ascribed to the vibrational mode of OH− groups of the HAp [33,34]. The absorption band that was located around 3600–3200 cm^−1^ corresponded to the absorbed water, which is a characteristic of aqueous synthesis methods [35,36]. The inset (b) in Figure 2 shows a clear shift to lower wavenumber values in the absorption band located around 1025 cm^−1^ due to the increased surfactant concentration. It is worth mentioning a similar behavior for the lattice parameters a and b, which slightly increased as a function of the surfactant concentration (see XRD section); the lattice parameter might have influenced the bond length, resulting in the shift of the absorption band located around 1025 cm^−1^ toward lower wavenumbers [37].

### 3.2. Morphological Studies

**Figure 3:**
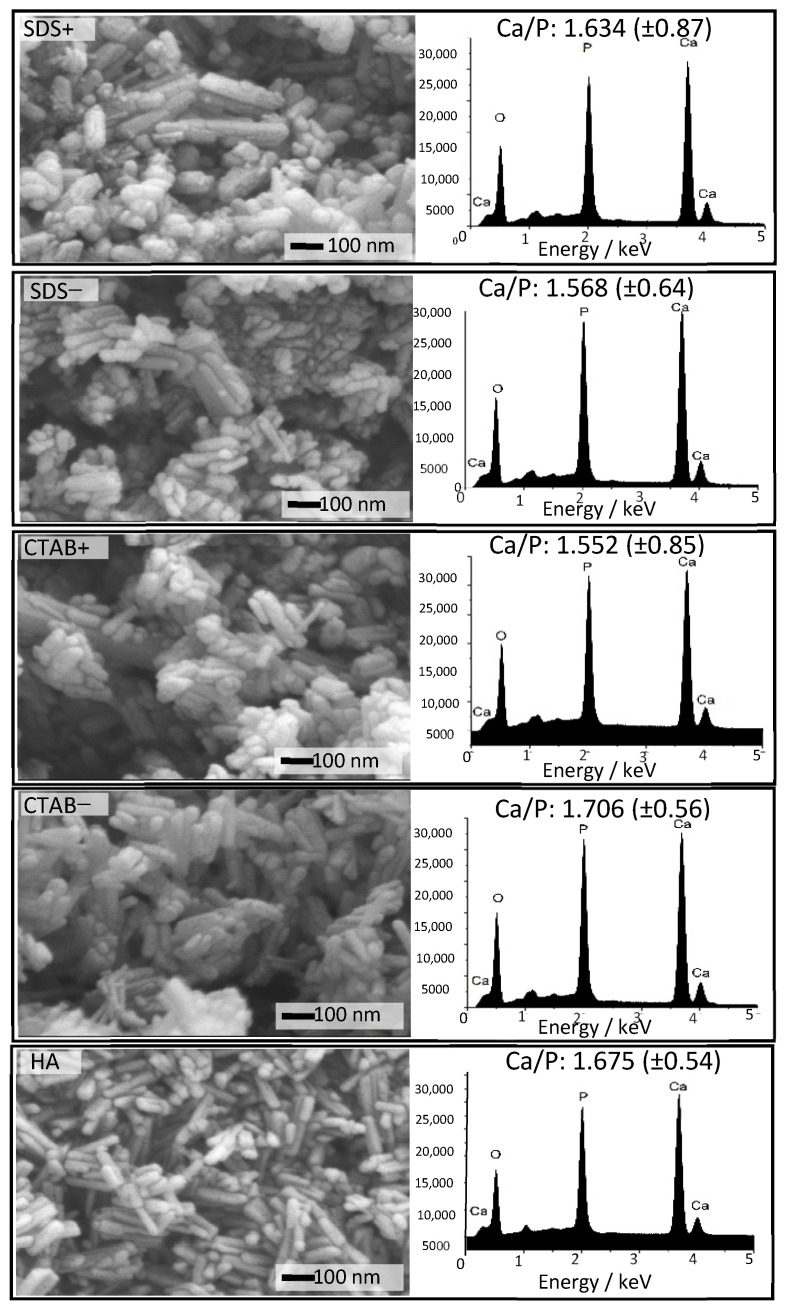
Representative SEM micrographs and EDS spectra of the HA, CTAB−, CTAB+, SDS−, and SDS+ powders.

**Figure 4:**
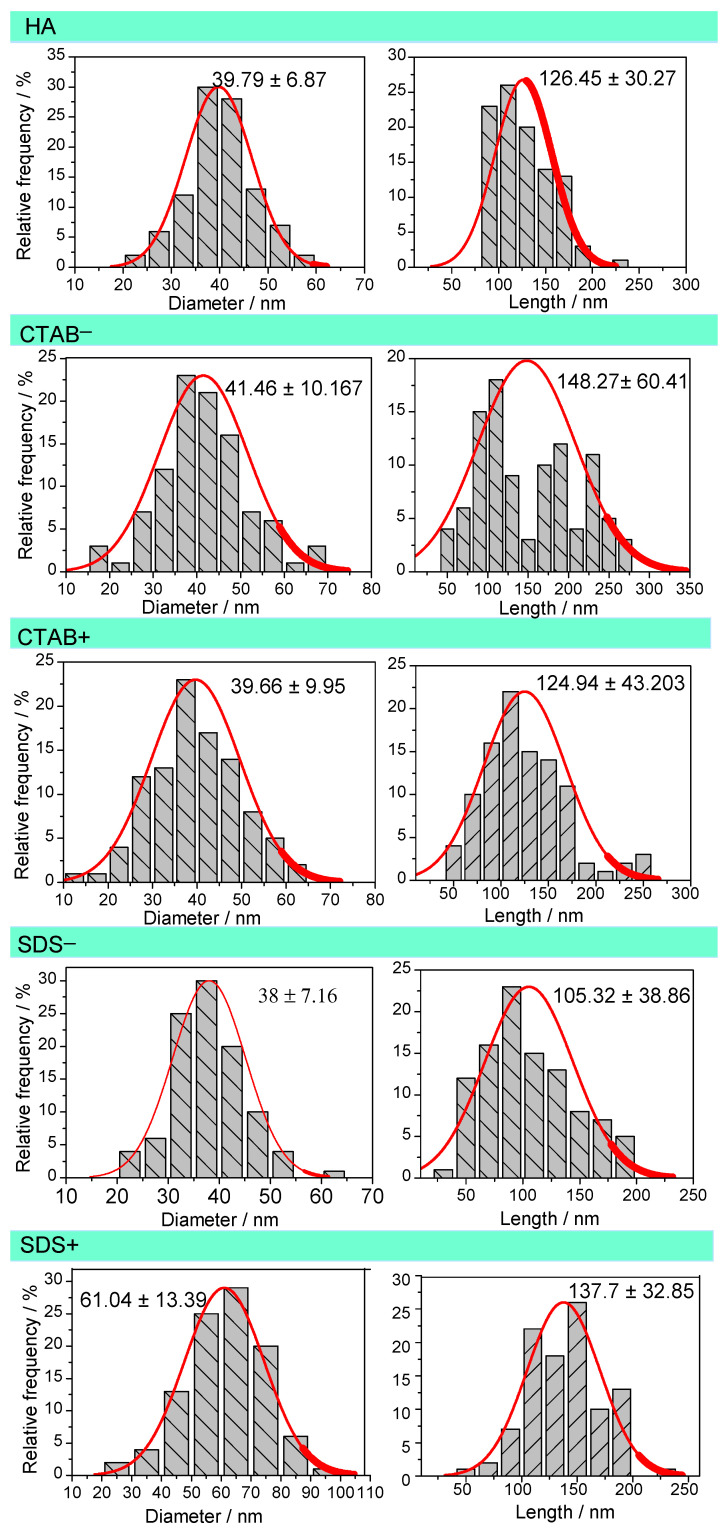
Diameter and length distributions of the HA, CTAB−, CTAB+, SDS−, and SDS+ samples. The numerical values report the mean and standard deviation of 100 measurements.

Scanning electron microscopy (SEM) is an important structural characterization method for studying the morphology evolution of powders. Figure 3 shows the SEM images of the HAp powders. The powder morphologies mainly consisted of rods particles ranging from 65–200 nm in length and 30–70 nm in diameter (Figure 4). The length distribution showed that the CTAB− sample presented the highest average (148.27 ± 60.41 nm), while the lowest average was for the SDS− sample (105.32 ± 38.86 nm). The diameter distribution indicated that the SDS+ sample had the highest average (61.04 ± 13.39 nm), while the SDS− sample had the lowest average (38.7 ± 7.16 nm). The length of the rods showed a significant difference (*p* < 0.05) between the HA, SDS−, and CTAB− samples. The lengths of the rods did not change when the surfactant concentration increased to 5 mol% (SDS+ and CTAB+). On the other hand, the diameters of the rods showed a significant difference (*p* < 0.05) for the sample SDS+; this observation was very interesting since the surfactant concentration could only affect one dimension, i.e., either diameter or length. From this, it can be concluded that at 2.5 mol%, the anionic surfactant increased the length of the rods and the cationic surfactant reduced it. Only the SDS at 5 mol% increased the length and the diameter.

The L/D ratio is an important parameter since it is a good indicator of whether the surface areas of the nanoparticles change. The average L/D ratios of the samples were 3.27 ± 0.99, 3.38 ± 1.36, 3.81 ± 1.2, 2.88 ± 1.04, and 2.73 ± 0.91 for HA, CTAB+, CTAB−, SDS+, and SDS−, respectively. The statistical analysis showed that the CTAB+ did not present a significant difference (*p* < 0.05) when compared with the HA. These results demonstrated that SDS and CTAB at 2.5 mol% changed the L/D ratio. It is worth noting that the L/D ratio decreased using SDS and increased using CTAB (only at 2.5 mol% of surfactant). Zhao et al. [38] found a preferential aggregation in calcium carbonate due to the surfactant interaction. On the other hand, Sanosh et al. [39] proposed that the OH− concentration determines the growth rate and morphology of HAp particles. In this study, the Ca^2+^ and PO_4_^3−^ movements during the nucleation were restricted due to the pH value used in the synthesis (pH = 11). As a result, the use of surfactants did not affect the morphology.

An electron dispersive spectroscopy (EDS) analysis was carried out to increase the structural information of the HAp samples (Figure 3). The average quantification of the Ca/P ratio corresponding to different zones on the HAp samples revealed slight differences between the samples. The average Ca/P ratio had a high standard deviation (see Figure 3) because of the precision of the technique and quantifications (three spots). Thus, it is not feasible to state with confidence that SDS or CTAB increased or decreased the Ca/P ratio. The findings in this study only suggested that the Ca/P ratio might be affected by the surfactants. However, the Ca/P ratio modification using surfactants was elucidated by Hajimirzaee et al. [40]. They observed a general trend toward an increased Ca/P ratio following the addition of surfactants (polyethylene glycol, poly(ethylene glycol)-block-poly(propyleneglycol)-block-poly(ethylene glycol), polyvinyl alcohol, polyoxyethylene sorbitan monolaurate, hexadecylamine, polyacrylamide), and a slight increase for CTAB. 

The Ca/P ratio was not a function of surfactant concentration since the surfactants normally present a critical micelle concentration (CMC); thus, a concentration higher or lower than CMC has a different behavior due to the surfactant that promotes the reduction of the interface energy. All the samples in the 1.55–1.67 range for Ca/P crystallized in the hexagonal structure of HAp (see Figure 1). The SDS− and CTAB− samples showed the largest L/D ratio variation between them; as a result, these samples, including the HA sample, were used to evaluate the antibacterial activity and dermal irritation and make a comparison with the HA sample.

### 3.3. Antibacterial Activity

**Figure 5:**
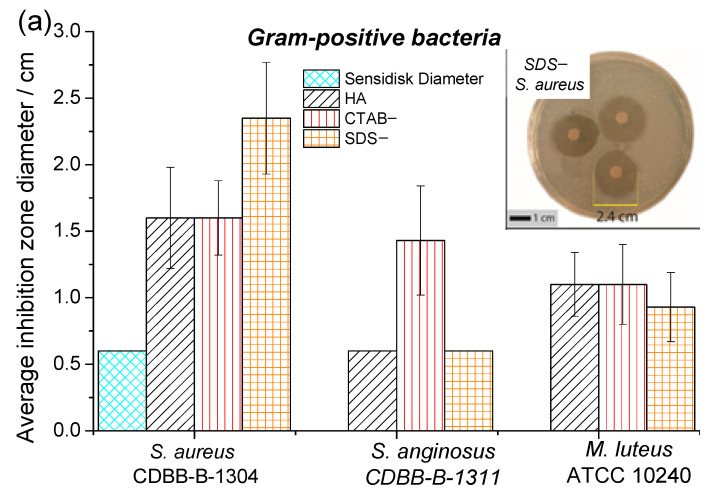

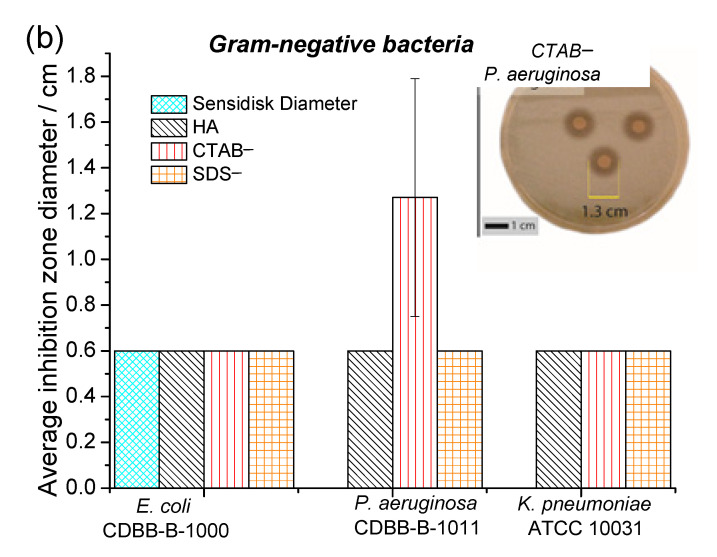
Inhibition zone diameter of HA, CTAB−, and SDS−, powders: (**a**) Gram-positive bacteria and (**b**) Gram-negative bacteria.

The antibacterial activity of HA, SDS−, and CTB− against Gram-positive and Gram-negative bacteria was evaluated using the disk diffusion assay. For this, the diameter of the inhibition zone (DIZ) (Figure 5a), which reflects the magnitude of the microorganism susceptibility, was measured; susceptible strains exhibit a larger diameter of the inhibition zone, resistant strains exhibit a smaller diameter of the inhibition zone [41,42]. Two possible mechanisms were associated with the antibacterial activity; (1) particle–bacteria interaction, where particles might penetrate the bacterial cell affecting the production of intracellular ATP, which interrupts the process of DNA replication; (2) surface area and surface charge of the particles such that when particles adhere to the cell membrane, they change the permeability [43].

Figure 5a shows that all samples presented an inhibition zone against *M. luteus* and *S. aureus*; this finding is very interesting given that *Staphylococcus aureus* is the main bacteria that is responsible for causing osteomyelitis. CTAB− displayed antibacterial activity against S. *anginosus* and *P. aeruginosa*, while their counterparts did not (see Figure 5b). This effect could be related to the surface area of the sample since CTAB− presented the higher L/D ratio and, therefore, exhibited a higher surface area, in addition to the structural change promoted by the synthesis condition (the use of CTAB as surfactant). Chen et al. [44] conducted a functionalization study of HAp particles with functional groups (amine group, carboxylic group, or methyl group) to change the HAp surface charges. They demonstrated that positively charged HAp particles could be more easily absorbed by the cells compared to negatively charged HAp particles of the same size. These results elucidate the possibility of changing the antibacterial activity using surfactants, even if the morphology of the HAp powders does not change [45].

### 3.4. Dermal Irritation

During the study, no clinical signs of disease were observed in the rabbits, where neither erythema nor necrosis was found at the inoculation sites. The papules formed in the inoculated dermal regions with the HAp solutions and the negative control were reabsorbed in 10 min after inoculation. All the rabbits presented papules at the inoculation sites after one hour of the positive control administration (Table 2). At 24 h, the sizes of the papules decreased at the inoculation sites of the positive control; edemas were observed in all rabbits that were inoculated with the HA, SDS−, and CTAB− solutions. At 48 h, only one rabbit that was treated with the HA sample did not present edema. At 72 h, four rabbits, namely, one inoculated with HA, one with CTAB−, and the others with SDS−, presented small palpable nodules at the inoculation site; similar findings were reported by Carlo et al. [46]. On the other hand, Kwon et al. evaluated dermal irritation caused by hydroxyapatite scaffolds in rabbits. They used a subcutaneous injection method and an exposure of 24, 48, and 72 h; the rabbits presented no significant biological reaction [47]. Thus, the dermal irritation findings in this research also suggest the viability of using HAp as a bone substitute.

Sepulveda et al. [48] evaluated synthetic hydroxyapatite HAP-91^®^ as a dermal filler; they showed that this intradermal implant tends to encapsulate itself and does not cause signs of toxicity but only presents an initial inflammatory response.

**Figure 6:**
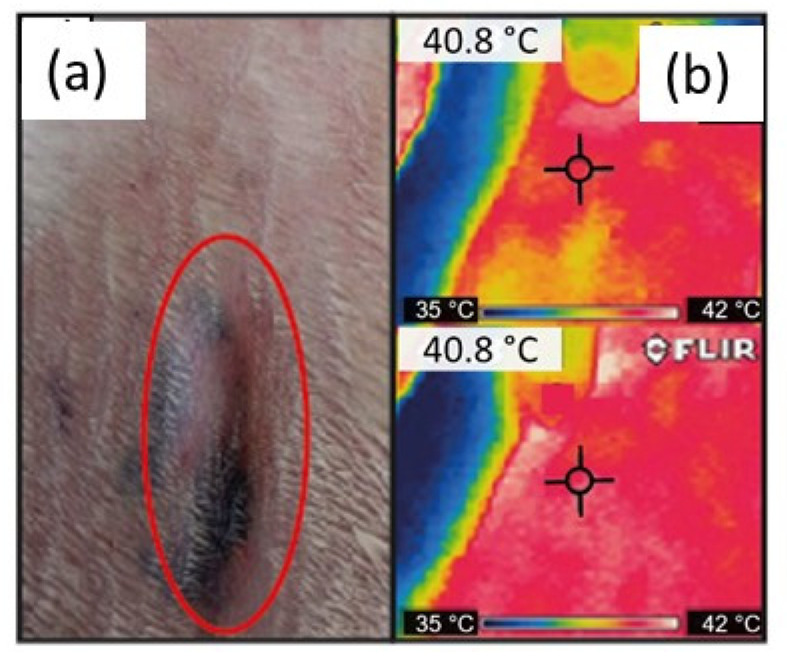
(**a**) Induration reactions at the site of inoculation observed at 72 h post inoculation; (**b**) thermography image.

In order to detect an increase in temperature at the inoculation sites of the rabbits during the intradermal reaction test; the temperature at the time of inoculation and 1, 24, 48, and 72 h post inoculation in the intradermal injection area was measured. For this purpose, infrared thermography, which is a modern, safe, and non-invasive alternative, was employed. This technique accurately detects a huge range of temperatures [49]. It is well known that skin temperature is an indicator of an animal’s health since it is related to local blood circulation and metabolism [50]. After one hour post inoculation, thermography images indicated an increase in the skin temperature at all the inoculation sites, which might have been due to the stress caused by handling at the time of the intradermal injection; the normal temperature in rabbits is 38.3 °C [51]. Ludwig et al. [52] demonstrated that stress situations in rabbits cause an increase in body temperature. One hour after inoculation, an increase in temperature was observed at all inoculation sites (Table 3 and Figure 6); this could be explained because one of the cardinal signs of inflammation is heat, which is caused by the inflammatory reaction due to the presence of the HA, SDS−, and CTAB− samples. At 24 and 72 h post inoculation, no increase in temperature was observed at the inoculation sites in the rabbits (Table 3).

## 4. Conclusions

Hydroxyapatite nanopowders were successfully obtained using a surfactant-assisted hydrothermal method. CTAB and SDS promoted a larger crystallite size of the hydroxyapatite powders. The use of SDS and CTAB modified the I_002_/I_112_ ratio, which produced different crystal orientations among the HAp powders. The lattice parameters a and b of the hexagonal phase increased when using SDS and CTAB. A low concentration of SDS and CTAB (2.5 mol%) was needed to modify the L/D ratio of the HAp powders. Samples with a higher L/D ratio increased the antibacterial activity. HA, SDS−, and CTAB− caused a reversible minimal edema response.

## Figures and Tables

**Table 1:** Lattice parameters, FWHM of the (002) peak, crystallite size, I_002_/I_211_, and L/D ratio of the hydroxyapatite samples.

Samples	Lattice Parameter (nm)		FWHM of (002) (Grades)	Crystallite Size (nm)	I_002_/I_211_
	a = b	c			
HA	9.42 ± 0.009	6.842 ± 0.041	0.371	23.827 ± 1.65	0.639 ± 0.073
CTAB+	9.424 ± 0.034	6.835 ± 0.048	0.272	34.027 ± 1.308	0.823 ± 0.048
CTAB−	9.447 ± 0.012	6.846 ± 0.011	0.301	29.717 ± 1.854	0.763 ± 0.024
SDS+	9.421 ± 0.026	6.854 ± 0.021	0.203	47.3 ± 1.188	0.8 ± 0.062
SDS−	9.43 ± 0.022	6.838 ± 0.076	0.293	30.347 ± 1.227	0.739 ± 0.025

The averages and standard deviations of the lattice parameter, crystallite size, and I_002_/I_211_ ratio were computed using three repeated measurements. L/D ratio was obtained using 100 measurements, where the length and diameter from a particular particle were selected. The FWHM value corresponding to the (002) peak in Figure 1c only represents the values but the average crystallite size was obtained from three repeated measurements.

**Table 2:** Inoculation parameters after the positive control administration.

Sample	Rabbit	1 h			24 h			48 h			72 h		
		C+	T	C−	C+	T	C−	C+	T	C−	C+	T	C−
CTAB−	24	++++p	0	0	++p	+e	0	+p	+e	0	0	+n	0
	25	++++p	0	0	++p	++e	0	0	+e	0	0	0	0
HA	23	++++p	0	0	++p	++e	0	+p	0	0	0	0	0
	26	++++p	0	0	++p	++e	0	++p	+e	0	0	+n	0
SDS−	27	++++p	0	0	++p	++++e	0	0	+e	0	0	++n	0
	28	++++p	0	0	++p	+e	0	0	+e	0	0	+n	0

p—papule, e—edema, n—small palpable nodule. The degree of edema papule and small palpable nodule of skin reaction was graded as 0, +, ++, +++, or ++++.

**Table 3:** Determination of the thermal profile of the intradermal inoculation sites.

Time (h)	Group				
	C ^(+)^	HA	C ^(−)^	CTAB−	SDS−
0	39.6 ± 0.6	39.2 ± 0.8	39.3 ± 0.8	39.7 ± 0.7	39.1 ± 0.6
1	41.2 ± 0.2	41.7 ± 0.2	41.2 ± 0.1	40.9 ± 0.2	41 ± 0.3
24	37.3 ± 0.4	37.2 ± 0.4	37.05 ± 0.6	37.4 ± 0.5	37.1 ± 0.6
72	37.9 ± 0.5	37.4 ± 0.6	38.2 ± 0.4	38.3 ± 0.3	37.5 ± 0.4

## Data Availability

Not applicable.
